# Predictive Ensemble Decoding of Acoustical Features Explains Context-Dependent Receptive Fields

**DOI:** 10.1523/JNEUROSCI.4648-15.2016

**Published:** 2016-12-07

**Authors:** Izzet B. Yildiz, Nima Mesgarani, Sophie Deneve

**Affiliations:** ^1^Group for Neural Theory, Laboratoire de Neurosciences Cognitives, Département d'Etudes Cognitives, Ecole Normale Supérieure, 75005 Paris, France, and; ^2^Department of Electrical Engineering, Columbia University, New York, New York 10027

**Keywords:** auditory cortex, Bayesian, decoding, encoding, explaining away, predictive coding

## Abstract

A primary goal of auditory neuroscience is to identify the sound features extracted and represented by auditory neurons. Linear encoding models, which describe neural responses as a function of the stimulus, have been primarily used for this purpose. Here, we provide theoretical arguments and experimental evidence in support of an alternative approach, based on decoding the stimulus from the neural response. We used a Bayesian normative approach to predict the responses of neurons detecting relevant auditory features, despite ambiguities and noise. We compared the model predictions to recordings from the primary auditory cortex of ferrets and found that: (1) the decoding filters of auditory neurons resemble the filters learned from the statistics of speech sounds; (2) the decoding model captures the dynamics of responses better than a linear encoding model of similar complexity; and (3) the decoding model accounts for the accuracy with which the stimulus is represented in neural activity, whereas linear encoding model performs very poorly. Most importantly, our model predicts that neuronal responses are fundamentally shaped by “explaining away,” a divisive competition between alternative interpretations of the auditory scene.

**SIGNIFICANCE STATEMENT** Neural responses in the auditory cortex are dynamic, nonlinear, and hard to predict. Traditionally, encoding models have been used to describe neural responses as a function of the stimulus. However, in addition to external stimulation, neural activity is strongly modulated by the responses of other neurons in the network. We hypothesized that auditory neurons aim to collectively decode their stimulus. In particular, a stimulus feature that is decoded (or explained away) by one neuron is not explained by another. We demonstrated that this novel Bayesian decoding model is better at capturing the dynamic responses of cortical neurons in ferrets. Whereas the linear encoding model poorly reflects selectivity of neurons, the decoding model can account for the strong nonlinearities observed in neural data.

## Introduction

The response properties of cortical neurons are typically characterized by their receptive fields (RFs) (Hubel and Wiesel, 1968; Theunissen et al., 2000). The underlying assumption is that each neuron acts as a feedforward filter of its stimulus, somewhat independently from other neurons. However, in addition to thalamic inputs, cortical neurons receive both excitatory and inhibitory intracortical inputs, strongly shaping their spontaneous and sensory-evoked activities (Lamme et al., 1998; Braitenberg and Schüz, 1998; Lee and Winer, 2008).

Several studies have shown that auditory spectrotemporal RFs (STRFs) can dynamically change depending on the stimulus characteristics (Theunissen et al., 2000; Valentine and Eggermont, 2004; Rabinowitz et al., 2011; Schneider and Woolley, 2011). This has led to more complex encoding models that incorporates contextual effects and nonlinearities into the RF analysis (Ahrens et al., 2008; Rabinowitz et al., 2012; David and Shamma, 2013; Willmore et al., 2016). Here, we hypothesize that the nonlinearities and context-dependency of neuronal responses can be described by a Bayesian network model with static decoding filters where neurons collectively and interactively aim to reconstruct their stimulus. Interactions between neurons allow for the recognition of sensory features that represents the input. Central to this inference mechanism is a basic reasoning argument (Wellman and Henrion, 1993) stating that a highly probable justification can “explain away” the stimulus, suppressing other possible interpretations. In particular, a sensory feature that is decoded (or explained away) by one neuron is not explained by another. Explaining away implies that selectivity of one neuron cannot be understood in isolation, outside of the context of other neurons representing overlapping stimuli. If one naively looks at the linear relation between the stimulus and the neural response (i.e., RF), this representation systematically changes and dynamically reshaped by surround stimuli. Instead, recovering the underlying fixed decoding filters can give a more stable description for the selectivity of each neuron. It can also provide conceptual motivation for why context-dependent, adaptive, gain-control models, such as (Ahrens et al., 2008; Rabinowitz et al., 2011, 2012), improve prediction performance over linear encoding models.

The main goal of this work is to introduce new methods to investigate the characteristics of this stable selectivity in auditory neuronal data. We start with a simple normative approach to model the structure and dynamics of the auditory system. In this architecture, auditory neurons are considered as predictors rather than filters of their input, as they collectively aim at inferring the underlying causes of the sensory stimuli (Smith and Lewicki, 2006; Lochmann et al., 2012). We approximate the decoding filters of auditory neurons from the primary auditory cortex of ferrets and use these filters in our Bayesian model to explain and predict the dynamics of neural responses better than an equivalent encoding model. The stimulus can be reconstructed from model responses as accurately as it can be reconstructed from the neural responses, and much more accurately than it can be reconstructed from the responses of the encoding model. This suggests that auditory neurons collectively represent the auditory scene (thus optimizing the capacity of subsequent layers to linearly reconstruct the stimulus from the neural responses), rather than responding to the auditory signal with fixed, independent RFs (which would require highly nonlinear decoding). Finally, we show that the predictive fields (PFs) learned from the statistics of speech are highly similar to neurons' actual decoding filters and that explaining away plays a crucial role in recovering a diverse set of features that fully represent the sensory environment.

## Materials and Methods

### 

#### A generative model of sounds

To be able to perceive a complex sensory scene, we need a schema that explains the observed stimulus by describing its underlying causes (Friston, 2005); for instance, hearing a knock on the door indicates a visitor hitting the surface of the door with the knuckles of his fist, which is not directly observed. This schema, so-called generative model, is a crucial ingredient for inference as it describes the internal hypothesis about the origin of the stimulus (Dayan et al., 1995; Friston, 2010). The presumed goal of the sensory system is to optimize this internal generative model until it can accurately explain the sensory data.

**Figure 1.**
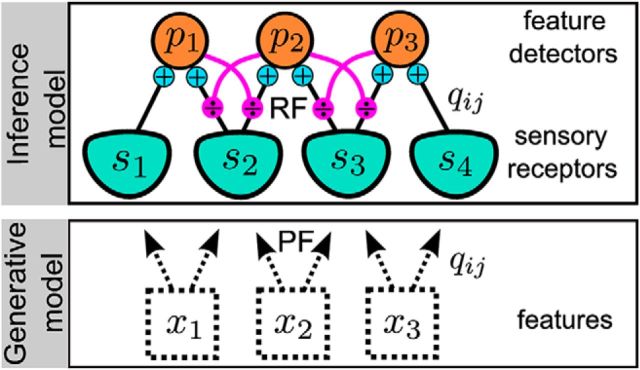
Inference model and its response properties. A schematic for the generative and inference models. Time-dependent features *x_i_* in the environment are encoded in the activity of feature detector neurons *p_i_* through sensory receptors, *s_i_*. Feature detectors receive input through feedforward (black solid) connections *q_ij_* (PF), which are also used to recurrently predict the stimulus (magenta connections). Feedforward and recurrent activity form the RF of neurons, which is different from the underlying selectivity (PF).

The generative model we consider (Deneve, 2008) (see Fig. 1, bottom) assumes that auditory events correspond to the coactivation of particular patterns of frequency. These events start and stop randomly, and combine linearly to generate the mean auditory signal. This auditory signal is corrupted by signal-dependent noise, as commonly observed in sensory neurons.

In more technical terms, elementary features correspond to variables *X* = {*x_j_*} that randomly switch on (*x_j_*(*t* − 1) = 0, *x_j_*(*t*) = 1) or off (*x_j_*(*t* − 1) = 1, *x_j_*(*t*) = 0) at each time step *t*, with constant probabilities *r*^on^ and *r*^off^. Each feature contributes to the resulting stimulus *S* by activating the sensory receptor responses *s_i_*. More precisely, the base firing rate *q_i0_* of a receptor *s_i_* increases by *q_ij_* ≥ 0 as a result of the presence of feature *x_j_*. We call the effect of a feature *x_j_* to the set of all receptors the PF of that feature, that is, *q_ij_* ≥ 0, *i* = 1 … *M* where *M* is the number of receptors. Sensory receptors produce noisy (Poisson) spike trains forming the likelihood as follows:

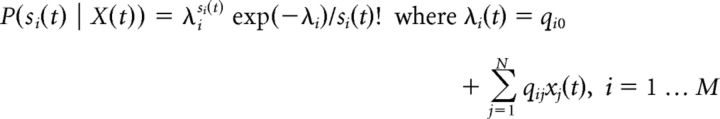


Without loss of generality, we remove the “baseline” firing rates *q_i0_* from the equations below since it is equivalent to having an additional hidden feature that is always “on”; that is, let:

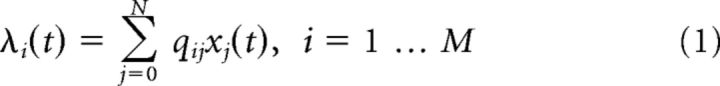
 where *x*_0_(*t*) = 1.

This likelihood together with the prior probabilities *r^on^* and *r^off^* forms the generative model. Throughout the manuscript, the stimulus will be in the form of frequency-time representation of sounds (spectrogram) where each frequency channel covers a small portion of the whole range from ∼100 Hz to ∼8 kHz. *s_i_*(*t*) represents a sensory receptor receiving input from the *i*-th frequency channel.

#### Bayesian inference and neural implementation of explaining away

The inference model computes the posterior probability of hidden features, *P*(*x* | *s*), by using the Bayes rule: *P*(*x* | *s*) = *P*(*s* | *x*)*P*(*x*)/*P*(*s*). For conceptualizing the inference model, we introduce the “feature detector units,” which keep track of the probability of *x_j_* being present at time *t* denoted by *p_j_*(*t*) = *P*(*x_j_*(*t*) = 1 | *s*(0 → *t*)), where *s*(0 → *t*) represents the input received from all receptors from time *0* up to *t*. Feature detectors can be thought as the auditory cortical neurons, and sensory inputs are either the stimulus (in its spectrographic form) or neural responses from a lower-level computational step.

We focus on the log-odds ratio of the hidden features as follows:

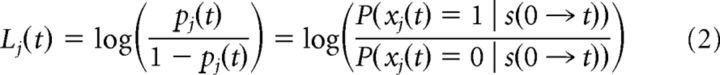
 Using the Bayes rule, the inference can be performed in an iterative process as follows:

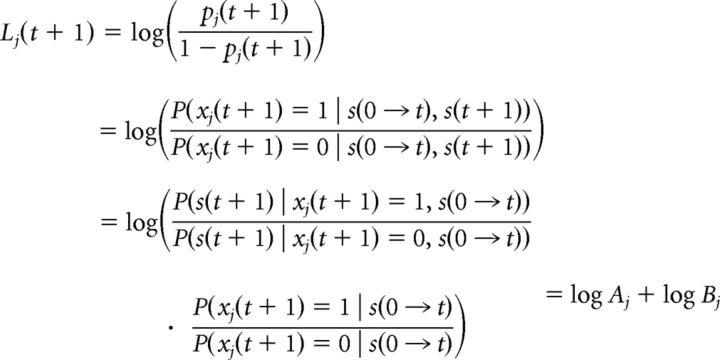
 The terms in *A_j_* represent the ratio of the likelihood of observations, and the terms in *B_j_* represent the ratio of priors. Because of the Markov property, the state of the object *j* at *t* + 1 depends only on its current state. The probability of *x_j_* being in an on-state at time *t* + 1 is possible either by being on at time t and staying on or switching from off to on (similarly for the probability of being off at time *t* + 1). Therefore, we can write the following:

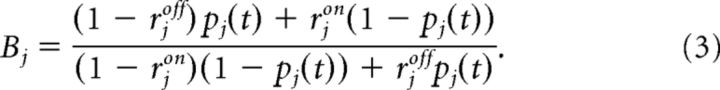
 Since each receptor unit, *s_i_*(*t*), is assumed to be independent from other receptors and its own history, the ratio of likelihoods can be written as follows:

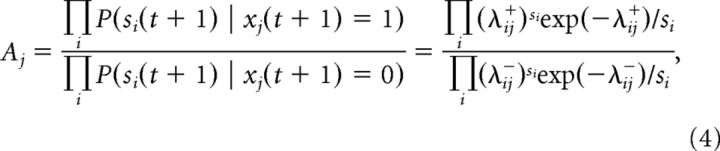
 where λ_*ij*_^+^ = ∑_*k*=0,*k*≠*j*_^*N*^ *q_ik_ x_k_*(*t* + 1) + *q_ij_*, that is, it is the λ*_i_* given in Equation 1 with the condition that *x_j_*(*t* + 1) = 1 and λ_*ij*_^−^ = ∑_*k*=0,*k*≠*j*_^*N*^ *q_ik_ x_k_*(*t* + 1), that is, it is the λ*_i_* with *x_j_*(*t* + 1) = 0.

The exact inference would require the computation of 2*^N^* feature configurations, and it is computationally expensive even for small models. Therefore, during the computation of likelihood of *x_j_*(*t* + 1), we use a mean-field approximation and replace all other hidden features with their expected values *p_k_*(*t*) from the previous time step: λ_*ij*_^+^ = ∑_*k*=0,*k*≠*j*_^*N*^ *q_ik_ p_k_*(*t*) + *q_ij_*, and λ_*ij*_^−^ = ∑_*k*=0,*k*≠*j*_^*N*^ *q_ik_ p_k_*(*t*). Taking the logarithm of Equation 4 and simplifying, we obtain the following:

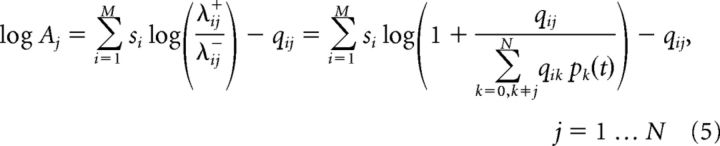
 This can be further approximated by ${\left[\sum_{i=1}^{M}s_{i}\left(t\right)\left(\frac{q_{ij}}{\sum_{k\neq j}q_{ik}p_{k}\left(t\right)}\right)-q_{ij}\right]}^{+}$ using log(*x* + 1) ≈ *x* for small *x*. Combining Equations 3 and 5, we conclude the iterative inference scheme for *L_j_*(*t* + 1). Once *L_j_*(*t* + 1) is known, we can use Equation 2 to compute *p_j_*(*t* + 1) = $\frac{e^{L_{j}\left(t+1\right)}}{1+e^{L_{j}\left(t+1\right)}}$.

We assume that the detector neuron's activity is proportional to the rectified log likelihood (log *A_j_*) rather than the posterior probability *L_j_*(*t*). The rationale behind this hypothesis is that sensory processing is inherently hierarchical. Thus, the output of the feature detector units will in turn be used as “sensory inputs” by similar units in later sensory stages. These higher-order detectors will themselves be integrators cumulating evidence for more complex (or more stable) features. To ensure self-consistency, sensory neurons should thus transmit new sensory evidence in favor of their preferred features *A_j_*, rather than sensory evidence cumulated over time *L_j_*. Therefore, we take (using log(*x* + 1) ≈ *x* for simplicity) the following:

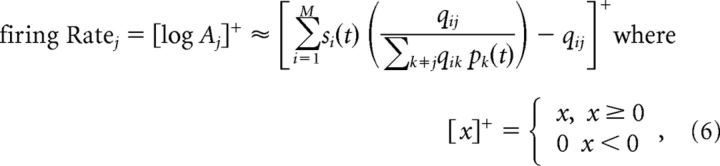
 Each detector unit can thus be interpreted as performing a weighted sum of sensory detector responses (see Fig. 1, black plain connections). However, the strength of connection from a sensory receptor to a feature detector is not fixed but divided by a prediction of this input by other detector units, that is, *ŝ_i_^j^*(*t*) = ∑_*k*≠*j*_ *q*_*i*0_ + *q_ik_ p_k_*(*t*) (see Fig. 1, magenta lateral connections). We can also interpret the feature detector as implementing divisive predictive coding as follows:

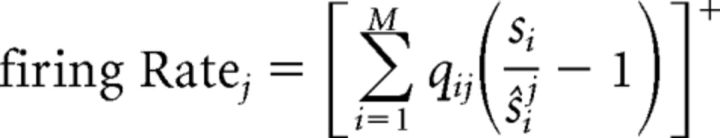
 where (*s_i_*/*ŝ_i_^j^*(*t*) − 1) is the prediction error from the *i*-th sensory receptor. In particular, when other detectors perfectly predict the receptor response, that is, *s_i_*(*t*) = *ŝ_i_^j^*(*t*), the contribution of this synapse is completely cancelled. Explaining away in a neural layer thus takes the form of a divisive inhibition targeting the neuron's inputs.

#### Model with no inhibition

Throughout the manuscript, we compare the responses of the intact model with the responses of the model without inhibition. In this version, feature detectors do not inhibit each other and the stimulus is processed in a feedforward fashion. The likelihood portion of the inference (Eq. 5) takes the form log *A_j_* = ∑_*i*=1_^*M*^ *s_i_*(*t*)log$\left(1+\frac{q_{ij}}{q_{i0}}\right)$ − *q_ij_*, and the rest of the inference remains the same.

#### Learning the PFs

Thanks to the normative approach, the PFs (i.e., the elementary, positive, and independent features composing the auditory scene) can be learned from the statistics of natural sounds (Lewicki, 2002; Smith and Lewicki, 2006). Once the online estimate of the *p_j_*(*t*),*j* = 1 … *N* has been inferred on a temporal window *t* ∈ [0,*T*] (Expectation step), the PFs *q_ij_* can be learned by maximizing the log likelihoods of observations given the expected values of the features as follows:

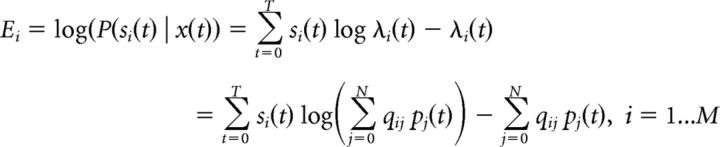
 The derivative at the maximum of the log likelihoods is zero as follows:

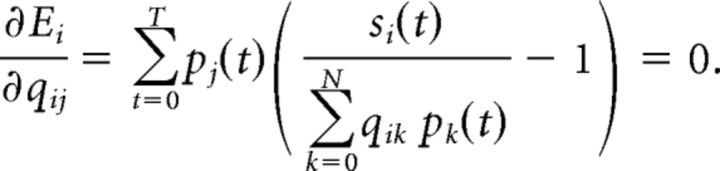


We solve this equation using stochastic gradient ascent in the simulations. We take *T* = 1 for online learning (at the same time-scale as inference). Since we defined *q_ij_* to be positive, if *q_ij_* becomes negative at a gradient ascent step, it is assigned to zero.

#### Neuronal recordings and stimulus

A detailed explanation of the experimental procedures was given by Mesgarani et al. (2009). Four adult, female, awake, and head-restrained ferrets were used in the neurophysiological recordings. The protocol for all surgical and experimental procedures was approved by the Institutional Animal Care and Use Committee at the University of Maryland and was consistent with National Institutes of Health Guidelines. Speech stimuli were taken from the Texas Instruments/Massachusetts Institute of Technology (TIMIT) database (Garofolo et al., 1993). Thirty different sentences (3 s, 16 kHz sampling) spoken by different speakers (15 men and 15 women) were used to sample a variety of speakers and contexts. Each sentence was presented five times during recordings. Speech spectrograms were binned at 10 ms in time and in 30 logarithmically spaced spectral bins between 125 and 8000 Hz. The total dataset consisted of 128 single-neuron recordings. We used *N* = 60 neurons throughout the paper satisfying a certain reconstruction accuracy threshold as described in more detail below.

#### Encoding and decoding filters

##### Encoding.

Encoding is the mapping from stimulus (spectrogram) to neuronal responses. We compute the encoding filters (STRFs; see Fig. 3*A* left) using normalized reverse correlation (Theunissen et al., 2001). Let *r̂*(*t*, *n*) = ∑*_i_* ∑_τ_*h_i_*(τ, *n*)*s_i_*(*t* − τ) where *r̂*(*t*, *n*) is the estimated response of neuron *n*, *h_i_*(τ, *n*) is the *i*-th frequency channel of its RF, τ is the delay, and *s_i_*(*t*) is the *i*-th frequency channel of the stimulus. *h*(τ, *n*) is approximated by minimizing *e_n_* = ∑*_t_*(*r*(*t*, *n*) − *r̂*(*t*, *n*))^2^ where *r*(*t*, *n*) is the average firing rate of neuron *n* over trials. It can be shown (Theunissen et al., 2001) that *h*(τ, *n*) = *C*_*SS*_^−1^*C_Sr_n__* where *C_SS_* is autocorrelation of the stimulus and *C_Sr_n__* is the cross-correlation between stimulus and neuronal response (for more explicit definitions, see Mesgarani et al., 2009). In practice, some form of regularization is required to prevent overfitting to noise. We used ridge regression where the regularization parameters are optimized using fivefold cross-validation in each training set.

##### Spectral RF (SRF) filters.

SRF (see Fig. 3*A*, right) is a reduced form of the full encoding filters (no time dimension). To compute the SRF for each neuron, we computed the normalized reverse correlation while we fix the delay. We used fivefold cross-validation in each training set to optimize the regularization parameters. To find the right delay between recordings and SRF predictions (see Fig. 4*A*), we shifted the recordings by 10 ms from 0 to 200 ms and reported the maximum correlation coefficient. This is done for all competing models.

##### Decoding.

Decoding is the mapping from neuronal responses to stimulus (spectrogram) (Bialek et al., 1991; Stanley et al., 1999; Mesgarani et al., 2009). These filters (see Fig. 3*B*, left) can be computed in a similar manner: Let *ŝ_i_*(*t*) = ∑*_n_* ∑_τ_ *g_i_*(τ, *n*)*r*(*t* − τ, *n*) where *g_i_*(τ, *n*) is the contribution of neuron *n* with delay τ to the reconstruction of the *i*-th frequency channel of the stimulus, *ŝ_i_*(*t*). For each *i*, *g_i_*(τ, *n*) is approximated by minimizing *e_i_* = ∑*_t_*(*s_i_*(*t*) − *ŝ_i_*(*t*))^2^. Then *g_i_*(τ, *n*) can be collected into a single structure *g*(τ, *n*) that forms the decoding filter for neuron *n*. It can be shown that *g_i_*(τ, *n*) = *C*_*RR*_^−1^*C_RS_i__* where *C_RR_* is the autocorrelation of neuronal responses and *C_RS_* s the cross-correlation between responses and the stimulus (Mesgarani et al., 2009). Similarly, we used ridge regression where the regularization parameters are optimized using fivefold cross-validation in each training set.

##### Extracted PFs from decoding filters.

Similar to the procedure with encoding filters, we can also extract simplified decoding filters that resemble the theoretically defined PFs. For each neuron *n*, we restricted the above analysis to a single time delay to recover frequency-only decoding filters. We used fivefold cross-validation in each training set to optimize the regularization parameters. To find the right delay between real stimulus and its model reconstruction (see Fig. 5*A*), we shifted the stimulus by 10 ms from 0 to 200 ms and reported the maximum correlation coefficient. This is done for all competing models.

##### Selection of neurons for analysis.

The dataset consists of 128 individual recordings from four animals. We combined the data from all animals into a single model assuming that similar stimulus selectivities would exist across animals. We were interested in a subset of neurons that carry relevant information about the stimulus. For this, we reconstructed the stimulus using each neuron's firing rates individually. If the reconstruction and actual stimulus had cross-correlation >0.2, the neuron was included for further analysis. This resulted in 62 neurons. We excluded two more neurons since they had completely negative decoding filters, which resulted in flat PFs.

#### Predicting neural activity

To use the model to get predictions for the neural activity (see Fig. 4), we obtained frequency-only decoding filters from the neural data as described in the previous section. We used these decoding filters as the PFs in the model. There were three free parameters that were adjusted globally to optimize the fit to neural data (*r^on^* = 1 and *r^off^* = 20 for each neuron and a global gain parameter for the output of the spectrogram). In all models (i.e., intact model) without inhibition and SRF, we found an optimum delay for each model prediction that maximizes the correlation coefficient to the neural responses on the training set. These delays were not changed during predictions over the test set. The dataset was divided into 10 equal portions over time, and all model prediction accuracies were averaged over 10-fold cross-validation process.

#### Transient and sustained responses

To identify responses after a sudden change of input to a neuron (see Fig. 6), we first identified the best frequency of each neuron from the maximum of its one-dimensional, frequency-only encoding filter. Then for each neuron, we searched for sudden increases in the spectrogram of the stimulus restricted to ±10 frequency bands around the best frequency and found the timings of the largest 150 local maxima in the derivative of the stimulus averaged over frequencies. For local maxima, we used findpeaks.m function in MATLAB (The MathWorks) with the condition that two consecutive local maxima are at least 200 ms apart. This gave us 150 events for each neuron. Transient responses are defined as the first 100 ms after such an event. Sustained responses are the following 100 ms after a transient response.

## Results

In the next few sections, we underline the main features of our normative model and provide evidence that auditory cortical neurons carry similar computations. We start with a simulation which shows that the RF (encoding filter) of a specific neuron can drastically change depending on the number of neurons in a network as a result of explaining away. Therefore, we expect that it would be challenging to predict the activity of a neuron without knowing the activity of other neurons in the network. Despite this difficulty, we wanted to test whether decoding filters could give a more reliable representation of neural activity. We approximated the decoding filters of several neurons in the primary auditory cortex of ferrets using linear regression and plugged these filters into our model as PFs. This allowed us to simulate neural activity that can accurately replicate the dynamics of actual neurons. We found that this simulated activity can reconstruct the stimulus much better than actual neural activity and the activity obtained from an encoding model. Finally, we show that PFs can be directly learned from the statistics of stimulus and that explaining away is crucial in obtaining variety of filters.

### Network effect on a single neuron

**Figure 2.**
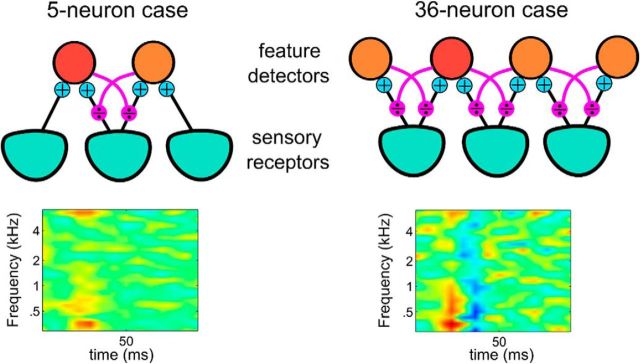
Changes in the RF of a neuron as neurons are removed from the network. The RF of a feature detector neuron (red) is computed from its responses in a small network of 5 neurons (left) and a larger network of 36 neurons (right). The stimuli for both cases were the same speech sentences from the TIMIT database. The responses of this neuron are shaped by the activity of all other neurons in the network that resulted in significant changes in its RF.

Despite the simplicity of the generative model (Fig. 1), the predicted unit responses (i.e., firing rates as given in Eq. 6) are highly nontrivial, and, in particular, cannot be understood in isolation, independently of other units. If, for example, we remove some of the PFs from the pool of available detectors (analogous to inactivating the corresponding sensory neurons), we find that the STRFs change (Fig. 2). This predicts that identifying the relevant features represented by sensory neurons and predicting their responses will be extremely challenging. In general, reaching perfect accuracy would require the simultaneous recordings of all neurons involved. Only a small subsets of all auditory neurons are recorded and included in this study, and they were not recorded simultaneously. Our primary goal in the next sections will thus be to clarify the functional and mechanistic implications of explaining away and accounting for particular aspects of the responses rather than focusing on precisely reproducing spike trains (as, e.g., in a GLM approach) (Paninski, 2004).

This normative approach illustrates three important predictions for auditory responses. First, if neurons are indeed involved in the optimal detection of overlapping auditory sounds, their responses will most likely be very poorly predicted by a linear encoding model. This is because the predicted responses are rendered highly interdependent by explaining away. Second, despite their apparent variability and absence of a fixed encoding model, the sound stimulus should be well reconstructed by a linear decoder applied to auditory responses. This linear decoder would indeed provide a better picture of neural selectivity than a linear encoding model. And third, even with the “right” decoding model, the normative model is unlikely to predict the neural responses with very high accuracy. This is because only a small subset of neurons are recorded and the rest are acting as “hidden variables” that affect the recorded neurons in unpredictable ways.

### Encoding versus Bayesian decoding models applied to neural data

**Figure 3.**
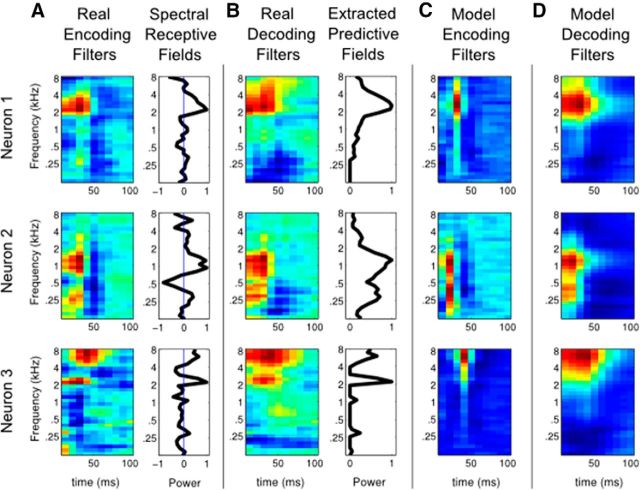
Encoding and decoding filters for real and model neurons. ***A***, Encoding filters of three auditory neurons (left) and their one-dimensional, frequency-only and normalized approximations: the SRFs (right). ***B***, Decoding filters of the same three auditory neurons (left) and their one-dimensional, frequency-only, and normalized approximations (right). They are much wider than the corresponding encoding filters in ***A***. ***C***, Inference model neurons, whose selectivities are given by the PFs in ***B***, have narrow encoding filters (similar to real encoding filters). ***D***, Decoding filters of the same model neurons are wider than the encoding filters in ***C*** and resemble the decoding filters of real neurons in ***B***.

In the auditory system, the response properties of neurons are usually characterized by STRF, which is the linear time-frequency filter best fitting the neuronal response when applied to stimulus (Aertsen and Johannesma, 1981). STRF describes how stimulus is encoded in the neuronal activity; therefore, it is also called the encoding filter (for examples, see Fig. 3*A*, left). Another way to look at the relationship between neuronal data and stimulus is to ask how stimulus can be decoded from the neuronal activity (Bialek et al., 1991). This method finds the linear mapping from the response of a population of neurons to the stimulus (see Materials and Methods), and the resulting frequency-time filter for each neuron is called the decoding filter (Fig. 3*B*, left).

Our Bayesian normative model suggests that context-dependent encoding filters could be replaced by the static decoding filters approximated from neuronal data, and then be used to predict the neuronal responses. To test this idea, we combined neuronal recordings from the primary auditory cortices of 4 ferrets listening to a 90 s speech stimulus from the TIMIT database (Garofolo et al., 1993) (see Materials and Methods). For each neuron, we computed a one-dimensional (frequency-only) approximation of the decoding filter that summarizes the full decoding filter (Fig. 3*B*, right). We call this filter the PF of the corresponding auditory neuron as it represents the contribution of the neuron in predicting the stimulus. Then, we plugged these PFs into the model as *q_ij_s* (no learning is involved). *r^on^* and *r^off^* rates were assumed equal among the neurons and chosen to optimize the fit between model and real neural responses. We interpreted the firing rate of each model neuron (Eq. 6) as a prediction for the firing rate of the corresponding real auditory neuron. As a baseline model, we also generated predictions from a model where inhibitory connections (i.e., explaining away) are removed and call it the model without inhibition. This altered model simulates a feedforward-only version of the intact model.

For comparison, we also computed a simplified encoding model for the neural data with the same basic structure. We reduced the full encoding filter of each neuron to one-dimensional (frequency-only) filter (Fig. 3*A*, right), which we call the SRF, convolved it with the stimulus, and rectified it to predict the neuronal activity.

### Performances of the encoding and Bayesian decoding models

**Figure 4.**
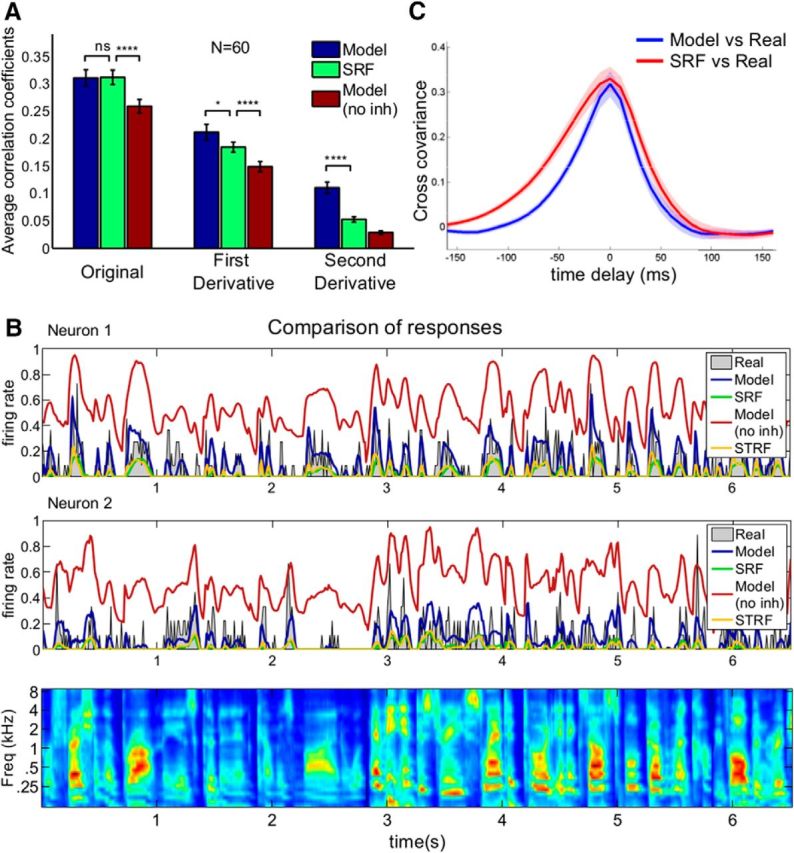
Prediction of neuronal activity by different models. ***A***, Comparison of average correlation coefficients (10-fold cross-validated) between the activity of 60 auditory neurons and their predicted responses by the intact Bayesian decoding model, SRF model, and Bayesian model without inhibition. Bayesian decoding model and SRF predictions are comparable (left; cc = 0.31 for both) and provide a better fit to real responses compared with the model without inhibition (cc = 0.26, *p* < 10^−10^, paired *t* test). For comparison, full STRFs reach a performance of cc = 0.38, significantly higher than the simplified encoding and decoding models. However, the first and second derivatives of the intact Bayesian decoding model responses (middle and right panels) fit the neural responses' first and second derivatives significantly better than the SRF predictions (*p* < 0.05 and *p* < 10^−6^, respectively). The Bayesian model better captures the detailed dynamics of the responses. Error bars indicate SEM. ***B***, Comparison of measured neural responses for two example neurons (gray in both panels) with their inference model predictions with or without inhibition (blue and red, respectively), SRF model prediction (green), and STRF prediction (yellow). ***C***, Cross-covariance between inference model versus real responses and between SRF versus real responses shows that model responses are temporally more precise than SRF responses. Shaded areas represent the 95% confidence intervals. *p < 0.05; ****p < 10^−4^.

The average cross-validated performances of the encoding and Bayesian decoding models in accounting for the neural firing rates are shown in Figure 4*A*. The model predictions and SRF predictions are comparable and better than the predictions of model without inhibition (Fig. 4*A*, left; *p* < 10^−10^, paired *t* test, *N* = 60 neurons). However, the ability of the Bayesian decoding model to capture the details of dynamic responses becomes more obvious when higher derivatives (first and second) of the neural responses and their predictions are compared (Fig. 4*A*, middle and right). The dynamics of the Bayesian model fits significantly better to the first (*p* < 0.05) and second (*p* < 10^−6^) derivatives of the responses compared with the encoding (SRF) model. This is the case even though the Bayesian decoding model is indirect, as it optimizes the reconstruction of the stimulus, not the neural responses as the encoding model does. For comparison, the full STRFs that take the temporal dimension into account (and have 750 free parameters each, even though these parameters are effectively decreased to ∼155 using ridge regression and only counting the parameters whose magnitude are >1 SD of the mean in each STRF) reach a performance of correlation coefficient = 0.38, significantly higher than the simplified encoding and decoding models with 31 (frequency channels and optimum delay parameters) and 34 (additional *r^on^*, *r^off^* and a global gain parameter for the PFs) parameters, respectively. In Figure 4*B*, we show the fits of the model (blue), model without inhibition (red), SRF (green), and STRF (yellow) predictions for the activity of two auditory neurons (gray). The model predictions are better at capturing the transient dynamics of neuronal responses than SRF and model without inhibition. Temporal precision of the model is higher in the entire dataset, as illustrated by the mean cross-covariance of the model versus real responses (Fig. 4*C*, blue) and SRF versus real responses (Fig. 4*C*, red).

**Figure 5.**
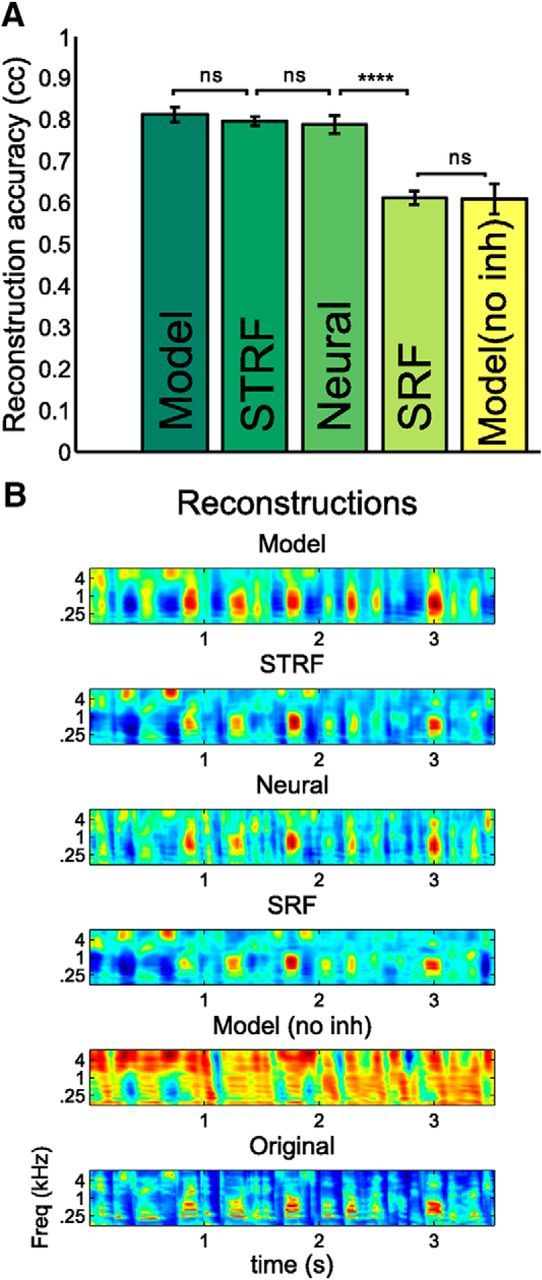
Reconstruction of stimulus by different models and recorded neural responses. ***A***, Comparison of reconstruction accuracies averaged over frequency channels (10-fold cross-validated) for the neural responses, SRFs, STRFs, and inference models. Intact model reconstruction (cc = 0.81) and STRF reconstructions (cc = 0.80) are as good as the reconstruction by the real responses (cc = 0.79), which is superior (*p* < 10^−10^) to the SRF and inference model without inhibition reconstruction (cc = 0.61). Error bars indicate SEM. *N* = 30 frequency channels, paired *t* test. ***B***, Short samples of reconstructed stimuli from model, SRFs, and neural responses as well as the original stimulus. ****p < 10^−4^

We also compared how well the stimulus can be reconstructed from the model neural responses using the full decoding filters (as in Fig. 3*B*, left) obtained from the real neural data. We took the responses of the Bayesian decoding model, SRF model, and model without inhibition and convolved them with the full decoding filters of the real neurons to reconstruct the stimulus. As summarized in Figure 5, we can observe that the Bayesian decoding model massively outperforms the encoding model. The stimulus can be reconstructed very accurately from the responses predicted by the Bayesian model (cc = 0.81). It is as good as the stimulus reconstructed directly from the neural data (cc = 0.79) or from the STRF predictions (cc = 0.80). If, on the other hand, we predict neural responses from their SRFs, and then try to reconstruct the stimulus, the performance is poorer (cc = 0.61) and clearly inferior to a reconstruction directly based on the neural data (*p* < 10^−10^, paired *t* test, *N* = 30 frequency channels).

In summary, the Bayesian decoding model outperforms the encoding model in how accurately it can represent the stimulus. Moreover, it is as good, if not better, than an encoding model of similar complexity when it comes to predicting neural responses, even when it is not directly fitted to those responses.

### Response dynamics are captured by explaining away

**Figure 6.**
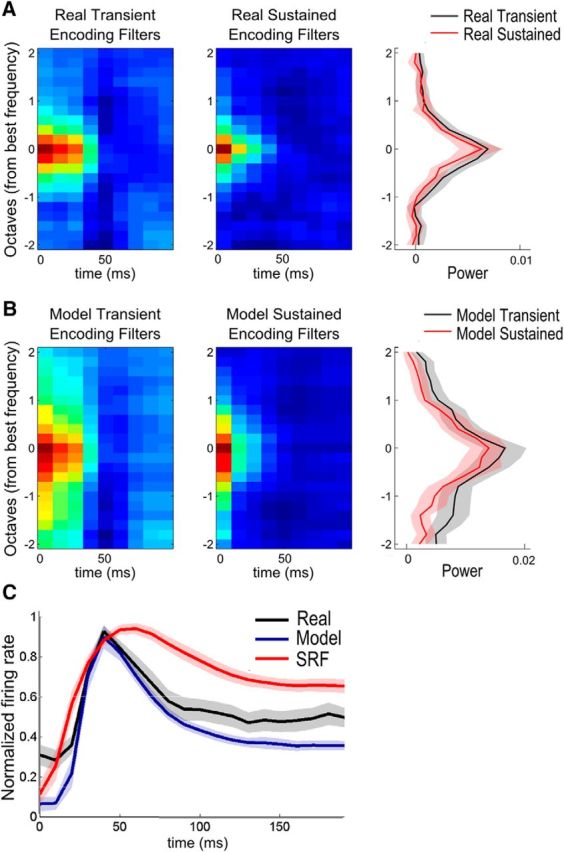
Transient and sustained encoding filters for real and model neurons (*N* = 60). We define an event as a sharp increase in the derivative of the stimulus around the best frequency of a neuron. Transient responses are defined as the responses 100 ms right after an event, and the sustained responses are the next 100 ms after transient responses. ***A***, The encoding filters of real neurons measured during transient (left) and sustained (middle) responses centered at their best frequencies and averaged over all neurons. Their one-dimensional (frequency-only) approximations (averaged over the first 30 ms of the full filters) are shown on the right. Encoding filters are much wider during transient responses. ***B***, The encoding filters of inference model neurons during transient (left), sustained (middle) periods, and their one-dimensional (frequency-only) approximations (right) show that the model neurons are able to capture the dynamic narrowing in the frequency selectivity of real neurons. ***C***, Mean response of real (black), inference model (blue), and SRF (red) responses after a sudden increase around the best frequency of each neuron. The response of each neuron is time-aligned according to the start of the event and the average 200 ms of activity for 60 neurons is plotted. Shaded regions represent the 95% confidence intervals.

We also observed other signatures of online inference, both in the neural data and in the Bayesian decoding model. We compared the transient and sustained responses of each neuron after the occurrence of a sudden transient in the stimulus (a sharp increase in the mean power averaged over frequency), such as the beginning of a new word or sentence (see Materials and Methods). Transient response is defined as the initial 100 ms after such an event, and sustained response is the next 100 ms after a transient response (for the average time course of responses after events, see Fig. 6*C*). Such dynamics are also consistent with the full STRF model. Immediately following these events, we observe a gradual sharpening of the RFs. Comparison of average RFs during transient and sustained responses for real neurons (Fig. 6*A*) shows the increased frequency selectivity as time progresses. A similar phenomenon is also replicated by the Bayesian model (Fig. 6*B*) due to the divisive competition between feature detectors. Following the occurrence of an event, many neurons are activated as they pool inputs from highly overlapping PFs. Transient response is strong but weakly selective. However, as more information is accumulated about the sound, some neurons become more confident than others about the presence of their preferred sound. This rise in divisive inhibition leads to decay in firing rates after transient response and sharpens the selectivity.

### PFs, not RFs, reflect neural selectivity

**Figure 7.**
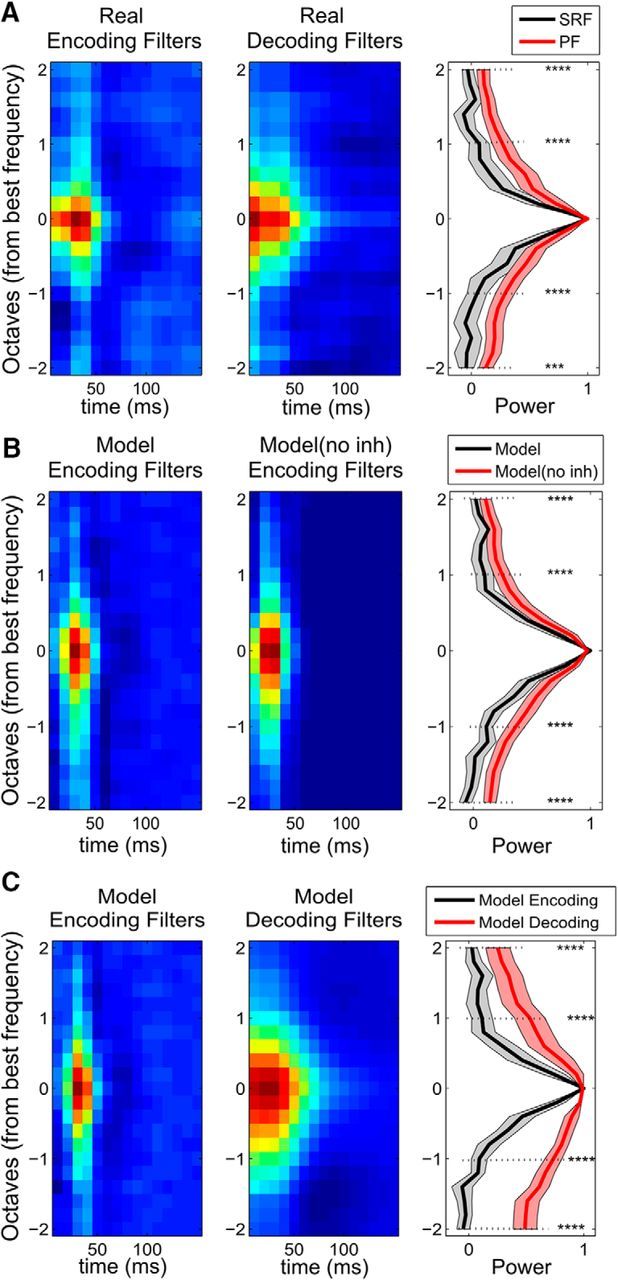
Summary of encoding and decoding filters for real and model neurons. ***A***, The encoding (left) and decoding (middle) filters of 41 neurons centered at their best frequencies and averaged. Their one-dimensional (frequency-only) approximations of SRF (encoding) and PF (decoding) are plotted on the right. The decoding filters are much wider than the encoding filters as quantified at ±1 and ±2 octaves from the best frequency. ***B***, Model neurons (*N* = 51) whose feedforward selectivity is extracted from the PFs (PFs in ***A***) have narrow encoding filters (left), similar to the real encoding filters, because of the explaining away effect. Inference model neurons with no inhibition have much wider encoding filters (middle) resembling closely the underlying selectivity (PFs in ***A***). ***C***, The comparison of encoding (left) and decoding (middle) filters of model neurons (*N* = 33) centered at their best frequencies and averaged. Their one-dimensional (frequency-only) approximations are plotted on the right. The decoding filters are much wider than the encoding filters as quantified at ±1 and ±2 octaves from the best frequency. ***p < 10^−3^. ****p < 10^−4^. Shaded regions represent the 95% confidence intervals.

Bayesian model neurons receive feedforward inputs pooled from their PFs (corresponding to the real decoding filters, Fig. 3*B*, right). However, their encoding filters (Fig. 3*C*) are reshaped by the competing activity of other model neurons. They are therefore narrower than the original PFs, with negative parts that are inexistent in the purely positive PFs (Fig. 3*B*, right vs Fig. 3*C*). The decoding filters of model neurons are wider and smoother than their encoding filters (Fig. 3*C* vs Fig. 3*D*). This phenomenon is also observed in the actual neural data. In particular, the measured encoding filters are markedly narrower than the corresponding decoding filters (Fig. 3*A* vs Fig. 3*B*), with additional negative subfields. To quantify this observation and present population trends, we plotted (Fig. 7*A*) the average encoding and decoding filters centered around the best frequency of each neuron (as long as the best frequencies of encoding and decoding filters are within ±3 frequency channels of each other, which was the case for 41 of 60 neurons). Model neurons reproduce this qualitative difference on average (Fig. 7*C*), that is, wide, positive decoding filters and narrow, positive/negative encoding filters.

If inhibitory effects between neurons are removed (model without inhibition), the encoding filters became wider (Fig. 7*B*, middle) and resembled the underlying feedforward connections (Fig. 7*A*, middle). Therefore, these effects are entirely due to explaining away. Observed differences between encoding and decoding filters of auditory neurons support the hypothesis of broad feedforward tuning (through PFs) that is narrowed by lateral inhibition (Wu et al., 2008; Isaacson and Scanziani, 2011; Moore and Wehr, 2013).

### Auditory neurons detect statistically independent auditory features

Next, we asked the question whether the shape of the real PFs (Fig. 3*B*, right) would naturally emerge from the model's learning algorithm and statistics of the stimulus. Because the generative model was based on independent Markovian statistics for different features, this is equivalent to asking whether neurons represent statistically independent features in their signal.

**Figure 8.**
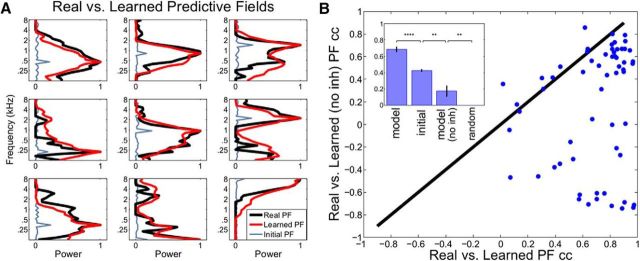
Real versus learned PFs (normalized) for nine neurons. ***A***, PFs obtained from real auditory neurons (black) can be learned by model neurons (red) with an Expectation-Maximization algorithm starting from an initial condition (light blue) with a small bump at the peak of auditory neurons' PFs. ***B***, Inhibition in the model is crucial for correctly approximating real PFs. The cc between real versus learned PFs in the intact model (horizontal axis, mean cc = 0.68) is significantly higher than the cc between real versus learned PFs in the model without inhibition (vertical axis, mean cc = 0.17). Inset, In addition to the inference model and inference model without inhibition (no inh) performances, we provide the mean cc between initial PFs (***A***, light blue) versus real PFs (initial, mean ≈ 0.43) and the mean cc between two arbitrary real PFs (random, mean ≈ 0). **p < 10^−2^; ****p < 10^−4^.

**Figure 9.**
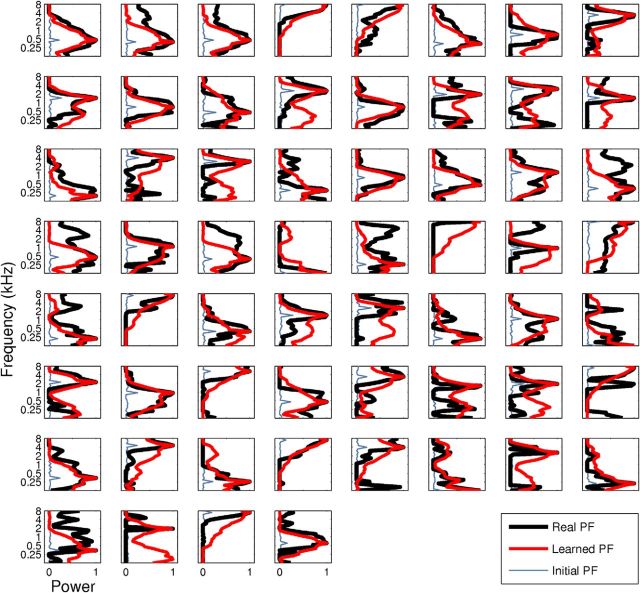
Real versus learned PFs (normalized) for all 60 neurons. PFs obtained from real auditory neurons (black) can be learned by most of the model neurons (red) starting from the initial conditions (light blue) as described in Auditory neurons detect statistically independent auditory features.

We used the same 90 s stimulus presented in the animal recordings to train the PFs of the model neurons. For each auditory neuron, we had a paired model neuron (*N* = 60). *r^on^* and *r^off^* rates were optimized globally to increase the reconstruction accuracy of the stimulus. As initial conditions, we used random PF connections for each model neuron with a small bump at the peak of its paired auditory neuron's PF (Fig. 8*A*, light blue curves). We then allowed the model to learn the PFs from the statistics of the speech stimulus using an Expectation-Maximization algorithm (see Learning the predictive fields). In Figure 8*A*, we show the PFs of 9 model neurons after learning (red curves) and the PFs of their paired real auditory neurons (for all 60 pairs, see Fig. 9, black curves). The structure of these PF pairs is highly similar (mean cc ≈ 0.68) and shows that the model can replicate the dynamics and selectivity of several auditory neurons only using the stimulus and rather flexible initial conditions. We compared this performance to two baseline conditions: (1) The cc between initial PFs (Fig. 8*A*, light blue) and real PFs (mean ≈ 0.43; Fig. 8*B*, inset: initial); and (2) the cc between two arbitrarily chosen real PFs (60 pairs compared, mean ≈ 0; Fig. 8*B*, inset: random), which were both significantly lower than the model performance.

To verify that a key ingredient of the model, explaining away, is necessary to recover the structure of PFs accurately, we used a secondary model with no inhibition between different feature detectors during inference. After learning, this model produced mostly repetitive set of PFs, and they were not as good as the intact model at replicating the real PFs (Fig. 8*B*; mean model cc = 0.68 vs mean model [no inh] cc = 0.17). If this comparison is limited to cc values that are positive for both model and model (no inh) (*N* = 40 neurons), the difference is still significant (mean model cc = 0.49 vs mean model [no inh] cc = 0.33, *p* < 0.001, paired *t* test). These results show that the competition between feature detectors is crucial for shaping their frequency selectivity and efficiently representing natural stimuli.

## Discussion

We presented a normative model where neurons interactively and collectively predict their input to optimally represent the underlying causes of their observations. A signature of this efficient representation is the competition between neurons representing similar stimulus features. The model predicts that feedforward inputs to a neuron (through PFs) are partially explained away by competing neurons which results in more narrow encoding filters (STRFs) compared with decoding filters. We also observed this signature in the analyzed auditory neurons (Figs. 3, 7). The dynamics of model responses are similar to neural data (Fig. 6) and can capture the activity better than a comparable feedforward model (Figs. 4, 5). In addition, we show that the underlying selectivity of some auditory neurons (PFs) can be accurately captured from the statistics of natural stimuli using the model's inference and learning algorithms (Figs. 8, 9). Despite originating from a functional hypothesis, the model is able to shed light on some fundamental mechanisms of sensory processing.

### Biological plausibility of the model

Identifying specific neural mechanism for inference and learning is not the main focus of this article. The model is normative, constrained only by optimality principles, and thus largely independent of its specific neural implementation. As an example, the algorithm presented here could be implemented in the auditory cortex by “projection” neurons (excitatory) representing the fractional prediction errors (*s_i_*/*ŝ_i_^j^* − 1) and inhibitory interneurons pooling the activity of projection neurons and inhibiting them divisively. In turn, our model neuron responses (the feature detector) could be obtained by summing the responses of these “projection” neurons. In any case, we note that the prediction errors (*s_i_*/*ŝ_i_^j^* − 1) used to update the activity of model units is zero on average, and is automatically kept as close to zero as possible by the inference algorithm. Thus, the experimentally observed balance between excitatory and inhibitory currents into cortical neurons (Wehr and Zador, 2003; Zhang et al., 2003; Okun and Lampl, 2008) could be interpreted as an imprint of an efficient network representing its input (Bourdoukan et al., 2012; Boerlin et al., 2013).

Moreover, explaining away provides a functional rationale for divisive normalization in which a neuron's response to a stimulus is given by its driving input divided by the summed activity of a pool of nearby neurons (Heeger, 1992; Heeger et al., 1996; Rabinowitz et al., 2011). This nonlinear phenomenon was proposed as a canonical computation for the brain because of the role it plays in the retina, visual cortex, olfaction, auditory system, and multisensory integration (Carandini and Heeger, 2011). Here, we argued that divisive normalization can naturally emerge in a neuronal system as a result of internal predictions through input-targeted inhibition. Normalization occurs between neurons that share similar feature selectivity, and it acts as a mechanism to reduce redundancy (Schwartz and Simoncelli, 2001; Lyu and Simoncelli, 2009).

In the present model, the PF of feature detectors can be learned by maximizing the log-likelihood, which requires only the input from presynaptic receptor cells and postsynaptic activity of feature detectors. Furthermore, these PFs can be learned in an online fashion. Learning could be implemented locally by Hebbian-like plasticity (Caporale and Dan, 2008) of the interconnection between projection neurons and inhibitory interneurons as well as the feedforward connections from projection neurons to feature detector neurons. With such algorithm, feature detectors can quickly adapt their selectivity to represent the recent statistics of stimulus, similar to the real sensory neurons in different modalities (Sharpee et al., 2006; Pérez-González and Malmierca, 2014).

### Encoding, decoding, and correlations

What accounts for the experimentally observed differences between encoding and decoding filters? As detailed in Materials and Methods, both encoding and decoding filters are based on the cross-correlation between the stimulus and neuronal activity. For encoding filters, this cross-correlation is normalized by the autocorrelation of stimulus, whereas for decoding filters it is normalized by the autocorrelation of neuronal responses (Mesgarani et al., 2009). Therefore, narrow encoding filters can be explained by the high autocorrelation of the natural stimuli (specifically speech) compared with the relatively low autocorrelation of neuronal responses. This relatively low autocorrelation cannot be explained entirely by neural noise because we used the average firing rate over five trials. We proposed explaining away as a possible reason for decorrelated neuronal responses. We showed that, if this inhibitory effect is removed from model neurons, their encoding filters become wider and resemble their decoding filters more closely (Fig. 7). Such extension of frequency tuning has been shown previously by blocking cortical inhibition pharmacologically (Chen and Jen, 2000; Wang et al., 2002) and supports the mechanism proposed in the model. This prediction could be further tested using existing optogenetic techniques: We would expect that silencing the inhibitory interneurons (such as parvalbumin-positive neurons) (Moore and Wehr, 2013) in the auditory cortex would result in broadening of the frequency tuning of the remaining excitatory pyramidal neurons. We would further predict that encoding and decoding filters of these excitatory cells would become more similar and their spike trains would get more correlated after suppression of inhibitory neurons.

### Relation to previous models

Predictive coding in the framework of Bayesian inference has been used successfully to explain classical and nonclassical RF properties in the visual domain such as end-stopping behavior (Rao and Ballard, 1999), dynamic adaptation to image statistics (Hosoya et al., 2005), and basic tuning properties and contextual effects (Spratling, 2010; Lochmann et al., 2012). However, it has been rarely used to explain neuronal phenomena in the auditory domain (Turner and Sahani, 2007; Ramirez et al., 2011; Yildiz and Kiebel, 2011; Wacongne et al., 2012; Yildiz et al., 2013) and, as far as we know, has never been used for predicting activity at a single-neuron level as achieved here.

Our functional approach assumes that single neurons represent specific events in the external world, and we deduce the neuronal mechanism from basic probabilistic principles. In contrast to previous functional approaches, such as efficient coding hypothesis (Olshausen and Field, 1996; Smith and Lewicki, 2006), we can learn and recognize stimuli in an online fashion, mimicking the biological auditory processing more closely, and we propose a plausible neural architecture with local, online learning rules for the parameters.

Our model is limited by its simple Markovian generative model. Markovian dynamics were chosen to introduce basic integrative properties in the model neurons while keeping the number of free parameters at a minimum. These dynamics could be improved with a more detailed generative model specific to the auditory system (Yildiz et al., 2013) and with inclusion of hierarchies representing different time scales and more complex features (Kiebel et al., 2008; Yildiz and Kiebel, 2011).

Top-down, normative modeling approach followed here is complementary to bottom-up, descriptive approaches, such as Linear-Nonlinear cascade (Chichilnisky, 2001), GLMs (Paninski, 2004), contextual RFs (Ahrens et al., 2008), gain control and adaptation (Rabinowitz et al., 2011; Rabinowitz et al., 2012; David and Shamma, 2013; Willmore et al., 2016), stimulus surprise (Gill et al., 2008), and maximally informative dimensions (Sharpee et al., 2004; Atencio et al., 2012). In particular, once these models are tuned for a specific type of stimulus, such as artificial or noise stimuli, they usually have to be retuned to explain neural responses with other types of stimuli. Instead, our model aims to identify the fixed selectivity of each neuron, independently of stimulus context and therefore presumably generalizable to different types of stimuli. Using protocols specifically designed to estimate these PFs rather than the stimulus-specific RFs of sensory neurons could unmask the underlying richness of these representations.
